# Cell-to-cell heterogeneity drives host–virus coexistence in a bloom-forming alga

**DOI:** 10.1093/ismejo/wrae038

**Published:** 2024-03-07

**Authors:** Nir Joffe, Constanze Kuhlisch, Guy Schleyer, Nadia S Ahlers, Adva Shemi, Assaf Vardi

**Affiliations:** Department of Plant and Environmental Sciences, Weizmann Institute of Science, 7610001 Rehovot, Israel; Department of Plant and Environmental Sciences, Weizmann Institute of Science, 7610001 Rehovot, Israel; Department of Plant and Environmental Sciences, Weizmann Institute of Science, 7610001 Rehovot, Israel; Department of Biomolecular Chemistry, Leibniz Institute for Natural Product Research and Infection Biology—Hans Knöll Institute, 07745 Jena, Germany; Department of Plant and Environmental Sciences, Weizmann Institute of Science, 7610001 Rehovot, Israel; Department of Plant and Environmental Sciences, Weizmann Institute of Science, 7610001 Rehovot, Israel; Department of Plant and Environmental Sciences, Weizmann Institute of Science, 7610001 Rehovot, Israel

**Keywords:** algal blooms, marine viruses, *Emiliania huxleyi*, host–virus interactions, resistance, single-cell analysis

## Abstract

Algal blooms drive global biogeochemical cycles of key nutrients and serve as hotspots for biological interactions in the ocean. The massive blooms of the cosmopolitan coccolithophore *Emiliania huxleyi* are often infected by the lytic *E. huxleyi* virus, which is a major mortality agent triggering bloom demise. This multi-annual “boom and bust” pattern of *E. huxleyi* blooms suggests that coexistence is essential for these host–virus dynamics. To investigate host–virus coexistence, we developed a new model system from an *E. huxleyi* culture that recovered from viral infection. The recovered population coexists with the virus, as host cells continue to divide in parallel to viral production. By applying single-molecule fluorescence *in situ* hybridization (smFISH) to quantify the fraction of infected cells, and assessing infection-specific lipid biomarkers, we identified a small subpopulation of cells that were infected and produced new virions, whereas most of the host population could resist infection. To further assess population heterogeneity, we generated clonal strain collections using single-cell sorting and subsequently phenotyped their susceptibility to *E. huxleyi* virus infection. This unraveled substantial cell-to-cell heterogeneity across a continuum of susceptibility to resistance, highlighting that infection outcome may vary depending on the individual cell. These results add a new dimension to our understanding of the complexity of host–virus interactions that are commonly assessed in bulk and described by binary definitions of resistance or susceptibility. We propose that phenotypic heterogeneity drives the host–virus coexistence and demonstrate how the coexistence with a lytic virus provides an ecological advantage for the host by killing competing strains.

## Graphical Abstract

**Graphical Abstract:**
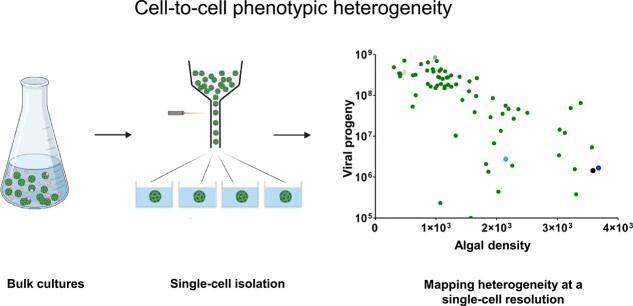

## Introduction

Algal blooms are ephemeral events of massive cell proliferation, serving as ecological hotspots of primary production and microbial interactions in the ocean [1]. Marine viruses are a major factor controlling algal blooms. By infecting their hosts, viruses reduce the host population size, reshape the bloom composition, and influence nutrient and organic carbon cycling [2]. Infection strategies of viruses vary from lytic infection to nonlethal infections such as lysogeny and chronic infection. Because of host replication dependency, host extinction is thereby an undesirable outcome. Nonlethal infections can ensure host survival as the virus can persist intracellularly without hindering cell division. In contrast, lytic viruses lead to rapid host cell death and virion release, preventing host–virus coexistence at the single-cell level [3-5]. This raises a key ecological question of how hosts and lytic viruses coexist, and how algal blooms reoccur throughout the years even though they are frequently terminated by lytic viruses. Host–virus coexistence has been reported at the population level for several taxa [6-9]; however, we still lack mechanistic understanding of this process, the dynamics at a single-cell resolution, and the effect of lytic infections on population heterogeneity, especially for ecologically relevant species.

The cosmopolitan coccolithophore *Emiliania huxleyi* and its specific virus, the *E. huxleyi* virus (EhV), are an important host–virus model system with significant ecological impact. *Emiliania huxleyi* forms vast annual spring blooms in temperate regions that can cover thousands of square kilometers in the ocean [10]. These large-scale blooms are often infected by EhV that acts as a dominant mortality agent causing bloom termination [11-13]. EhV is a large (ca. 180 nm in diameter), double-stranded DNA virus with a lytic life cycle and high burst size [14]. Despite its virulent nature, EhV does not lead to *E. huxleyi* extinction, as evidenced by the multi-annual cycle of bloom and demise. This dilemma becomes more complex when the virus is specialized on a bloom-forming alga, where host availability is limited to the high cell densities during ephemeral bloom events. This specialized strategy of EhV is very challenging in the long periods lasting many months in between blooms, especially with regard to the short half-life of virions in the aquatic ecosystem leading to a daily decrease in their infectivity [15-18]. It has been shown that *E. huxleyi* strains vary in their susceptibility to different EhV isolates and that most *E. huxleyi* strains are resistant to some, if not all, EhV isolates [14, 19]. This prominent occurrence of virus resistance among *E. huxleyi* strains suggests complex host–virus interactions that prevent the eradication of the virus over evolutionary time scales. Although *E. huxleyi* and its lytic virus have coexisted for thousands of years in the natural environment [20], the ecological and biological processes facilitating their coexistence are underexplored [21]. Previous studies suggest that *E. huxleyi*–EhV dynamics follow the continuous arms race model, whereby both players genetically adapt to overcome one another. A central manifestation for such coevolution is the large arsenal of auxiliary metabolic genes (AMGs) that are encoded by EhV and transcribed during infection. AMGs rewire the host cell metabolism and thereby facilitate the production of essential building blocks for new viral progeny. For example, the EhV genome encodes an almost complete sphingolipid pathway leading to the biosynthesis of virus-derived glycosphingolipids (vGSLs) during viral infection [22-24]. The viral genes are homologs to the corresponding host genes, indicating horizontal gene transfer as part of the evolutionary arms race between *E. huxleyi* and EhV [25]. In addition, *E. huxleyi* may evade viral attack by morphological and life-cycle changes within a small subpopulation of cells that promote resistance [26, 27]. Nevertheless, infection experiments in the lab typically end with one winner, either EhV or a resistant *E. huxleyi* strain [26-31]. We thus lack an experimental model system to study host–virus coexistence.

We aimed to investigate the occurrence of host–virus coexistence in the *E. huxleyi*–EhV model system, its effect on infection dynamics, and its link to host phenotypic heterogeneity. We examined the interaction between the susceptible *E. huxleyi* strain CCMP 2090 (hereinafter, *E. huxleyi* 2090), and the lytic virus strain EhV-201 in lab-based experiments. Specifically, we characterized a resistant *E. huxleyi* culture that recovered from viral infection, named *E. huxleyi* 2090-Rec, which maintained a parallel proliferation of both the host and virus. We isolated 74 clonal cultures that were derived from single-cell sorting of *E. huxleyi* 2090-Rec and phenotyped their susceptibility to viral infection. In contrast to an expected binary resistance or susceptibility in these clones, we revealed a wide spectrum of resistance levels across these single-cell isolates. These results highlight the cell-to-cell heterogeneity within host populations and provide a new perspective on the binary definition of resistance and susceptibility at the population level. We propose that the phenotypic plasticity of *E. huxleyi* is the driving force for establishing coexistence with its lytic virus and suggest how the multi-annual dynamic of *E. huxleyi* bloom and EhV bloom termination is sustained without host or virus extinction.

## Materials and methods

### Strains of *E. huxleyi* and EhV

The following *E. huxleyi* strains were obtained from the Roscoff Culture Collection or from the National Center for Marine Algae and Microbiota: CCMP2090, CCMP373, CCMP374, CCMP379, and RCC1216 (hereinafter: *E. huxleyi* 2090, 373, 374, 379, and 1216, respectively), as well as RCC6945, RCC6946, and RCC6955, which were isolated in 2018 from a mesocosm experiment in Bergen (Norway). The culture *E. huxleyi* 2090-Rec recovered from strain 2090 following infection with EhV-201. The *E. huxleyi* strains Rec-17, Rec-32, Rec-53, and Rec-97 were derived by single-cell sorting from *E. huxleyi* 2090-Rec. The *E. huxleyi* strains 2090-BD5 and 2090-2 were derived by single-cell sorting from *E. huxleyi* 2090. For viral inoculation, the following EhV strains were used: EhV-201, EhV-86, EhV-163 [14], EhV-ice01 [15] (hereinafter EhV-ice), EhV-M1 [32], and EhV-Rec, that were propagated on *E. huxleyi* as listed in Table S1.

### Culture maintenance and experimental conditions

Algal cultures were grown at 18°C with a 16:8-h light:dark cycle and a light intensity of 100-μmol photons m^−2^ s^−1^, provided by cool white light-emitting diodes. Cultures were diluted weekly at a ratio of 1:10 into fresh medium. The medium was composed of autoclaved and filtered seawater (FSW) supplemented with modified K/2 medium (replacement of organic phosphate with 18-μM KH_2_PO_4_) [33] and the antibiotics ampicillin (100-μg ml^−1^) and kanamycin (50-μg ml^−1^). For regular virus infection assays, algal cultures were grown in 50-ml flasks. For infection assays that included single-molecule fluorescence *in situ* hybridization (smFISH) sampling, cultures were grown in 650-ml flasks.

### Viral lysate preparation and algal culture inoculation

A fresh viral lysate was propagated 1 week prior to every infection assay. Each EhV strain was propagated with its respective algal host strain (Table S1). In brief, 1000 ml of an exponentially growing *E. huxleyi* culture at 1–2 × 10^6^ cells ml^−1^ was inoculated with EhV in a 5:1 virus-to-cell ratio. After 4 days, the viral lysate was filtered through a 1.2-μm pore size glass microfiber filter (grade GF/C, GE Healthcare Whatman) followed by a 0.45-μm pore size Nalgene Rapid-Flow filter unit (PES, Thermo Fisher Scientific) to remove cell debris, before concentrating and washing the virions by 100-kDa tangential flow filtration (Vivaflow 200, Sartorius). The concentrated virions were sterile-filtered through a 0.22-μm filter (PVDF, Millex-GV, Millipore) and stored in darkness at 4°C until the infection assay. For EhV-Rec, which coexists with the algal host *E. huxleyi* 2090-Rec, no inoculation was needed. EhV-Rec virions were filtered and concentrated as described above, however, without the final filtration through 0.22 μm to prevent the loss of virions that possibly occurred because of filter clogging by transparent exopolymer particles. For every infection assay, an exponentially growing algal culture at ~5 × 10^5^ cells ml^−1^ was inoculated at a 1:1 virus-to-cell ratio.

### Enumeration of algal cell abundance, cell death, and viral particles abundance

Algal cells and viruses were monitored by flow cytometry (Supplementary Text). In brief, living algal cells were identified by their chlorophyll fluorescence, whereas the fraction of dead algal cells was quantified following the staining with Sytox Green (Fig. S14A–C). Viral particles were fixed with glutaraldehyde and stained with SYBR Gold before flow cytometry analysis (Fig. S14D). Data analysis was conducted using CytExpert 2.4 (Beckman Coulter).

### Calculation of growth rate (*μ*), carrying capacity, and maximum viral production

The growth rate (*μ*) was calculated as $\mu =\ln \left({N}_2/{N}_1\right)/\left({t}_2-{t}_1\right)$, where *N_1_* represents the cell abundance at time 1 (*t_1_*), and *N_2_* represents the cell abundance at time 2 (*t_2_*) [34]. *t_1_* is the first day of the exponential growth phase of the untreated cultures, and t_2_ is the last day that a culture grows exponentially. The same calculation was conducted to estimate *μ* for the EhV-inoculated cultures. The carrying capacity (CC) is the highest cell abundance that each culture reached. The maximum viral production (MVP) represents the highest abundance of extracellular viral particles measured during each infection assay. All measurements were conducted for EhV-inoculated and non-inoculated cultures of each *E. huxleyi* strain in three biological replicates.

### Quantification of actively infected *E. huxleyi* cells using smFISH

To estimate the fraction of infected cells within an *E. huxleyi* population, we used smFISH with probes targeting the EhV major capsid protein (*mcp*) gene, as previously described [4]. In brief, a mix of 47 probes with a prob length of 20 nucleotides was designed to bind the *mcp* mRNA of EhV at different locations. Conjugation with the fluorophore tetramethylrhodamine allows the detection by flow cytometry (ex: 561 nm, em: 564–606 nm). Cells with >10^3^ A.U. signal area were enumerated as *mcp* positive cells. Aliquots of 30-ml algal culture were fixed with paraformaldehyde (1% final concentration) and incubated for 1 h at 4°C with agitation. Samples were centrifuged for 2 min, the supernatant was discarded, and the pellets were resuspended in 1-ml cryopreserving solution. To ensure contact of the cryopreservant with all fixed cells before freezing, samples were incubated for 1 h at 4°C with agitation. Samples were centrifuged, the supernatant removed, and the pellet stored at −80°C until hybridization. For hybridization, samples were thawed at room temperature, and chlorophyll was extracted by sequential resuspension in 70% and 90% ethanol (HPLC grade, J.T. Baker). Samples were treated with 500-μl proteinase-K (10-μg ml^−1^ final concentration, Ambion). Then, 50 μl of hybridization buffer (17.5% formamide) containing the *mcp* probes (0.1-ng ml^−1^ final concentration) were added to all samples for overnight incubation at 30°C in darkness. Lastly, samples were stained with DAPI (10-μg ml^−1^ final concentration), resuspended in 400-μl GLOX buffer, and analyzed by flow cytometry (FSC height threshold = 5 × 10^3^ A.U., SSC area threshold = 1 × 10^3^A.U.).

### Generation of monocultures by single-cell sorting and screening for viral resistance

To generate clonal cultures (herein monocultures) from *E. huxleyi* cultures, single cells were sorted using fluorescence-activated cell sorting with BD FACSAria III Cell Sorter (BD Biosciences). Cells were gated based on their optical properties (chlorophyll fluorescence ~5 × 10^4^ A.U., forward scatter ~5 × 10^4^ A.U.) and sorted into 200-μl fresh medium in 96-well plates. The well plates were wrapped with parafilm to prevent evaporation and incubated at 18°C and low light (~20-μmol photons m^−2^ s^−1^) with a 16:8-h light:dark cycle. After 4–8 weeks, wells that showed algal growth were transferred to new well plates and maintained by a 10-fold dilution into fresh medium every 3 weeks. To screen for viral resistance, the monocultures were transferred to two 96-well plates, one serving as an uninfected control and the other for inoculation with EhV and cultured at ~100-μmol photons m^−2^ s^−1^ (Supplementary Text).

### Quantification of lipid markers for viral infection using UPLC-HRMS

To assess the occurrence of virus-infected cells within *E. huxleyi* populations by the formation of virus encoded vGSLs, we used LC–MS-based untargeted lipid profiling as previously described [35]. In brief, cultures of *E. huxleyi* 2090-Rec, 2090-BD5, and 2090 with and without addition of EhV-201 were analyzed for their cellular lipid composition in three technical replicates (except 2090-BD5, for which two replicates were analyzed). Cultures were grown in 1000-ml FSW with modified K/2 medium, and ampicillin and kanamycin as described above. Samples of *E. huxleyi* 2090-Rec, 2090-BD5, and 2090 were collected during exponential growth phase, whereas samples of 2090 inoculated with EhV-201 were collected at 3 days postinoculation (dpi), during culture lysis. All samples (150–200 ml of each culture, equivalent to ~1–3 × 10^8^ cells per sample) were collected by vacuum filtration onto 1.6-μm pore size glass microfiber filters (grade GF/A, GE Healthcare Whatman), immediately plunged into liquid nitrogen, lyophilized to dryness, and stored at −80°C. Lipids were extracted with methyl tert-butyl ether with using glucosyl (β) ceramide d18:1/c12:0 as internal standard, and the dried extracts analyzed by UPLC-HRMS (Supplementary Text) [24, 36, 37].

### Enumeration of viruses in *E. huxleyi* 2090-Rec derived monocultures by qPCR

To verify if the single-cell isolates from *E. huxleyi* 2090-Rec produced viruses, we assessed EhV presence in the medium of the monocultures derived from *E. huxleyi* 2090-Rec using quantitative PCR (qPCR) and primers for the *mcp* gene of EhV. Aliquots of each monoculture were centrifuged, and 50 μl of cell-free supernatant were boiled for 20 min at 100°C to release DNA from virions. Samples were diluted in 120 μl of ultrapure water to prevent qPCR reaction inhibition by medium substances. The qPCR analysis was conducted as previously described [38], using 5′-acgcaccctcaatgtatggaagg-3′ (mcp1Fw) and 5′-rtscrgccaactcagcagtcgt-3′ (mcp94Rv) as primers. Concentrated EhV-Rec virions were used as positive control and FSW as negative control. All reactions were carried out in technical triplicates.

### Cocultivation experiments of two *E. huxleyi* strains

To assess the outcome of intraspecies competition, we cocultured pairs of *E. huxleyi* strains separated by a 1-μm membrane, allowing the free exchange of viruses, inorganic nutrients, and metabolites such as vitamins. One strain was inoculated into the wells of a 12-well plate and the second strain into Thincert inserts (Greiner). Cell abundances and virus production were monitored for both strains located within each vessel. Before sampling, cultures were resuspended with a serological pipette to counteract the sedimentation of cells and enhance medium exchange. The pore size of 1 μm allowed viruses to pass the membrane, thus, the enumerated virus abundances cannot be attributed to a single algal strain when both strains were susceptible. Experiments were conducted in three biological replicates. As a control, all strains were cocultured with themselves by inoculating them both in the wells and in the inserts (S12A–E).

### Statistical analysis

**Figure 1:**
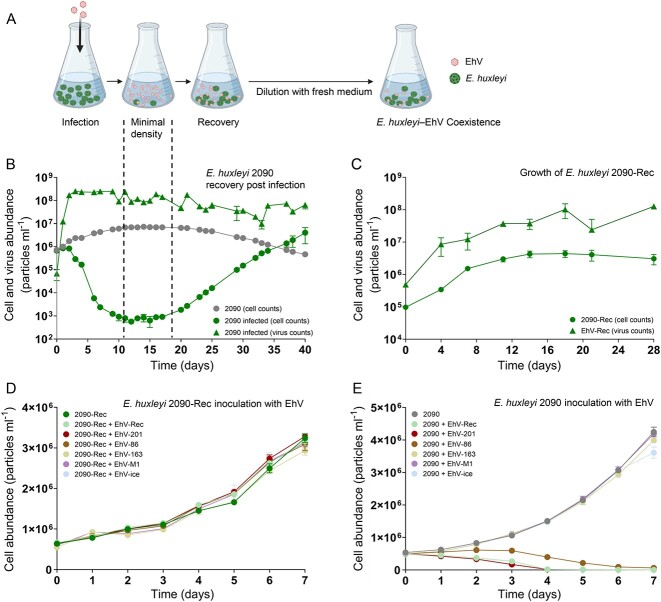
Coexistence of lytic viruses with a population of *E. huxleyi* cells recovered from viral infection. (A) Schematic representation of the experimental system. Upon viral infection, *E. huxleyi* populations decline to a small number of cells that survive and subsequently form a new population. This recovered resistant population proliferates in the presence of lytic viruses and produces virions over many generations. (B) Infection of *E. huxleyi* 2090 with EhV-201 led to the recovery of the resistant population *E. huxleyi* 2090-Rec in the presence of lytic viruses. (C) Concomitant proliferation of host cells and virions in cultures of the recovered coexisting *E. huxleyi* 2090-Rec following continuous sub-cultivation in fresh growth medium. (D) Growth of *E. huxleyi* 2090-Rec cells is not affected by inoculation with different EhV strains. (E) Growth of ancestral *E. huxleyi* 2090 cells upon inoculation with different EhV strains. Values are presented as mean ± SD (*n* = 3). Scheme was created with Biorender.com.

The significance differences in cell abundance and virus abundance of 2090-Rec treated with different EhV strains (Figs 1D and S3A) was calculated using one-way ANOVA with Šidák correction for multiple comparisons by comparing the mean of each treatment to the non-treated culture at 7 dpi. The significance of the differences in chlorophyll fluorescence and virus production between *E. huxleyi* 2090-Rec-derived monocultures and 2090-derived monocultures (Fig. 3B and C) was assessed using a two-tailed nonparametric Mann–Whitney test. The coefficient of variance (CV) was calculated as follows: CV = standard deviation/sample mean × 100. The correlation between chlorophyll fluorescence and virus production of *E. huxleyi* 2090-Rec monocultures was calculated by nonparametric Spearman correlation. The significance of the differences in growth rates and CC across all strains and treatments was estimated using two-way ANOVA with Tukey’s multiple pairwise comparisons (Figs S9 and S10). The significance of the differences in MVP was estimated using one-way ANOVA with Tukey’s multiple pairwise (Fig. S8). The effect of EhV-Rec on the growth of *E. huxleyi* 2090-BD5 was assessed using a two-tailed *t*-test to compare the cell abundances of *E. huxleyi* 2090-BD5 cocultured with *E. huxleyi* 2090-Rec (Fig. 4E) and *E. huxleyi* 2090-BD5 cocultured with itself (Fig. S12C) on day 7 of the cocultivation. All calculations were conducted using GraphPad Prism 9.3.1 (GraphPad Inc.).

## Results

### Development of a model system for studying host–virus coexistence

To test whether the microalga *E. huxleyi* and its specific virus EhV can establish coexisting populations under controlled culture conditions, the susceptible *E. huxleyi* 2090 was infected with the lytic viral strain EhV-201 (Fig. 1A). The infection dynamics were monitored for 40 days by counting host and virus abundances using flow cytometry (Fig. 1B). At 2–11 dpi, the host population showed a virus-induced decline of 99.9% of the cells (Fig. 1B). The population was not lysed completely and remained at a minimal cell density of ~10^3^ cells ml^−1^ for 6 days. At 20 dpi, the recovery phase started, characterized by increasing cell abundances that exceeded the initial host population density at 37 dpi. The host population decline in the first 3 dpi was coupled with high viral production (Fig. 1B) and up to 80% cell death at 6 dpi (Fig. S1). The recovered host population grew in the presence of substantial viral load (6 × 10^7^–1 × 10^8^ viral particles ml^−1^), suggesting the development of phenotypic properties that differ from the susceptible ancestor strain and provide resistance to viral infection. To examine virus resistance in the recovered host population, the cultures were diluted weekly into fresh medium, reaching a dilution of at least 10^8^, to minimize the virions of the viral lysate (Fig. 1A). Nonetheless, virus propagation (>1 × 10^8^ viral particles ml^−1^) was detected in parallel to algal cell growth in these cultures, reaching a maximum of 4 × 10^6^ cells ml^−1^ in the stationary phase (Fig. 1C). The emergence of a recovered resistant cell population, named *E. huxleyi* 2090-Rec (2090-Rec), together with the proliferation of a co-occurring virus, named EhV-Rec, suggests the development of a stable coexistence between *E. huxleyi* and EhV. We then examined whether EhV-Rec exhibits different infectivity toward *E. huxleyi* strains as compared with the ancestor EhV-201. The infectivity of EhV-Rec toward the susceptible *E. huxleyi* strains 2090, CCMP374 (hereinafter, 374), and RCC1216 (hereinafter, 1216), as well as the resistant strain CCMP379 (hereinafter, 379), was similar to EhV-201 (Fig. S2). Thus, EhV-Rec exhibited a similar host range and infection dynamics as EhV-201, suggesting that no major phenotypic differences exist between the virions at infection phase compared with the coexistence state. Furthermore, virus resistance in the recovered *E. huxleyi* population 2090-Rec was tested against the virus strains EhV-201, EhV-Rec, EhV-86, EhV-163, EhV-M1 [32], and EhV-ice [15], which differ in their host range and infection dynamics with the ancestral *E. huxleyi* strain 2090 (Fig. 1D and E). In contrast to the inoculation with 2090, none of these virus strains affected the proliferation of 2090-Rec cells (Fig. 1D*P*-value =  0.0931) or led to additional viral production (Fig. S3A) (*P*-value = 0.1207). When inoculated with EhV-201, EhV-Rec, or EhV-86, cultures of the ancestral *E. huxleyi* strain 2090 declined (Fig. 1E), accompanied by an increase in virus abundance (Fig. S3B) as previously shown for EhV-201 and EhV-86 [39-41]. Taken together, we observed similarities between the infection dynamics governed by the coexisting virus EhV-Rec, as compared with its ancestor strain EhV-201, and dissimilarities in the susceptibility of the coexisting host population *E. huxleyi* 2090-Rec, as compared with its ancestor strain *E. huxleyi* 2090. Therefore, we hypothesize that the generation of the host–virus coexistence state was driven by changes in the host population rather than in the virions themselves.

### Coexisting recovered populations are heterogenous and composed of subpopulations

**Figure 2:**
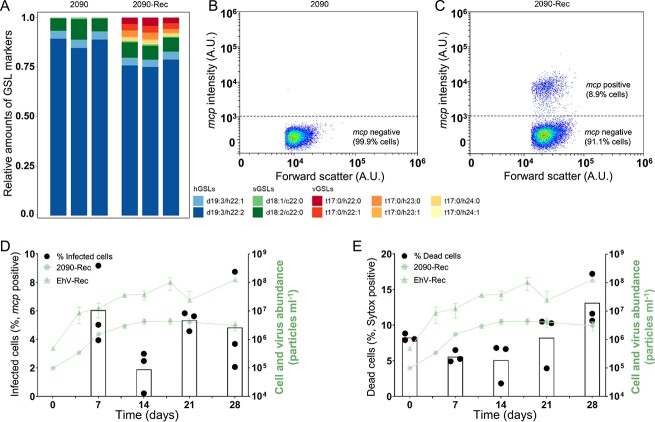
The recovered coexisting *E. huxleyi* 2090-Rec population comprises a subpopulation of virus-infected cells undergoing cell lysis. (A) Relative amounts of GSL markers (hGSLs, sGSLs, and vGSLs) in *E. huxleyi* 2090 and 2090-Rec. (B) Single-cell expression of the viral *mcp* gene using smFISH in the uninfected 2090 culture shows no expression of the viral *mcp* gene (*n* = 13 942). (C) Single cells from the recovered coexisting culture *E. huxleyi* 2090-Rec during late exponential growth (day 7). Among these cells 8.9% expressed the viral *mcp* gene (*n* = 17 612), using a threshold intensity of 10^3^ A.U. for the *mcp* probe. Boxplots depict the fraction of (D) infected cells (*mcp* positive cells, as quantified by smFISH) and (E) dead cells (Sytox positive) in cultures of *E. huxleyi* 2090-Rec throughout growth (left *y*-axis). Green lines depict counts of cells (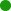) and viruses (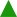) (right *y*-axis), as in Fig. 1C. Values are presented as mean ± SD (*n* = 3).

*Emiliania huxleyi*–EhV interactions have been assessed so far mainly by bulk measurements, assuming phenotypic homogeneity of the cells within a population. The observed coexistence of a resistant host population and a lytic virus suggests the co-occurrence of phenotypically diverse cells within the host population. We thus sought to assess the phenotypic heterogeneity within the *E. huxleyi* 2090-Rec population in comparison to a population of the ancestral strain *E. huxleyi* 2090 using established molecular and metabolic markers for viral infection in *E. huxleyi* [23, 36]. We applied lipid biomarkers (GSLs) that can inform about the phenotypic cell states of *E. huxleyi*, namely, uninfected cells (host-derived GSLs, hGSLs), susceptible cells (sialic acid GSLs, sGSLs), and virus-infected cells (vGSLs) [23, 36]. Exponentially growing cultures of *E. huxleyi* 2090-Rec were analyzed using a liquid chromatography-high-resolution mass spectrometry-based lipidomics approach and compared with infected and uninfected cultures of the ancestral strain *E. huxleyi* 2090. Cell cultures of the coexisting *E. huxleyi* 2090-Rec were comprised of hGSLs, sGSLs, and vGSLs similar to virus-infected *E. huxleyi* 2090, whereas uninfected cultures of the ancestral strain comprised only hGSLs and sGSLs (Figs 2A and S4). The detection of vGSL in *E. huxleyi* 2090-Rec cultures indicates the presence of a subpopulation of infected cells, and because of the stable growth of the *E. huxleyi* 2090-Rec cultures, we assume the population is composed of another subpopulation of uninfected resistant cells. To further quantify these subpopulations in *E. huxleyi* 2090-Rec, we used smFISH, which enables quantification of viral transcripts at a single-cell resolution and informs about the fraction of infected cells in a population [4]. Flow cytometry was used to detect and enumerate cells with a positive fluorescence signal using probes that target mRNA transcripts of the viral *mcp* gene (Fig. 2B and C). In *E. huxleyi* 2090-Rec, the fraction of cells that were actively infected (*mcp* positive) was on average 2%–6% throughout the exponential and stationary growth (Fig. 2D), whereas the fraction of dead cells was 5%–13% (Fig. 2E). In comparison, uninfected cultures of *E. huxleyi* 2090 showed only *mcp* negative cells (Figs 2B and S5) and 2%–4% of dead cells (Fig. S1). These results demonstrate that the coexisting *E. huxleyi* 2090-Rec is a heterogeneous population comprising at least two subpopulations with contrasting phenotypes: one being resistant and one infected. Whereas bulk cell abundance measurements suggest that the *E. huxleyi* 2090-Rec population is overall resistant, the presence of vGSLs, *mcp* positive cells, and elevated levels of dead cells indicate that a small fraction of cells in this resistant recovered population is virus-susceptible and undergoing lytic viral infection.

### Mapping population heterogeneity in resistance to viral infection at a single-cell level

**Figure 3:**
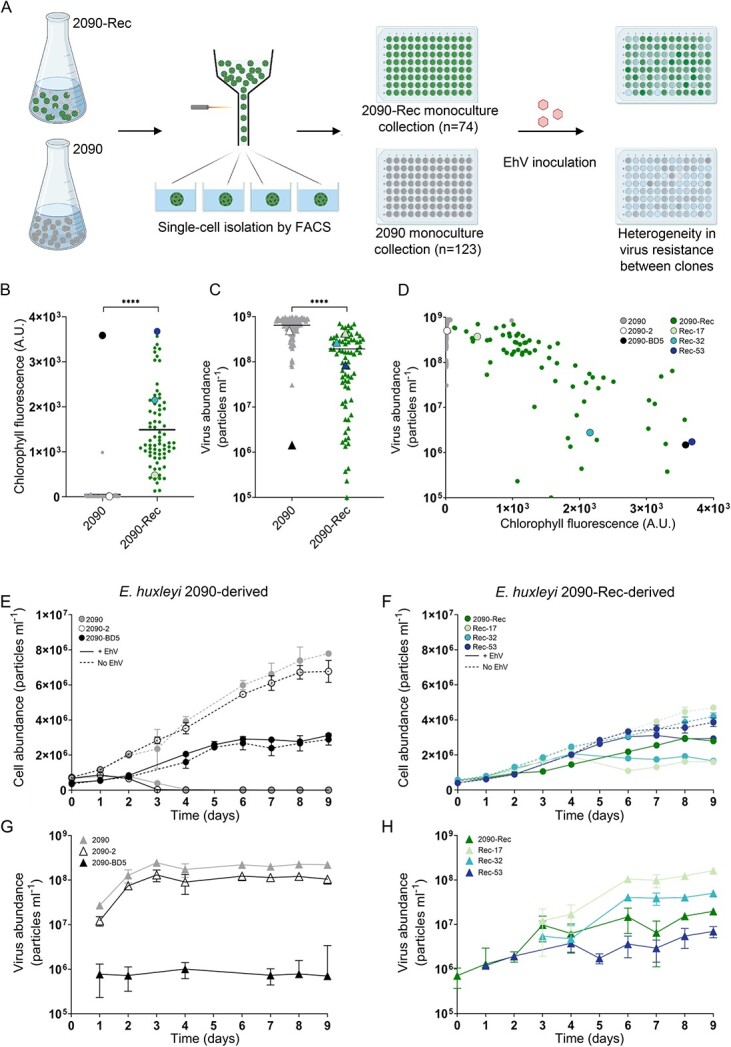
Cell-to-cell heterogeneity in resistance to viral infection within the coexisting *E. huxleyi* 2090-Rec population. (A) Experimental procedure of the single-cell sorting and phenotyping for viral resistance. Single cells were sorted from susceptible strain *E. huxleyi* 2090 and coexisting culture *E. huxleyi* 2090-Rec, and the derived monocultures were inoculated with EhV-201. The resistance phenotype of each monoculture was characterized at 6 dpi. (B) Chlorophyll fluorescence of monocultures derived from *E. huxleyi* 2090 (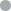) and 2090-Rec (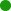), as a proxy for cell growth and survival during viral infection. The horizontal line represents the mean value. (C) Virus abundance in monocultures derived from *E. huxleyi* 2090 (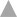) and 2090-Rec (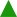). The horizontal line represents the mean value. (D) Virus abundance as a function of chlorophyll fluorescence in monocultures derived from *E. huxleyi* 2090 and 2090-Rec. Monocultures with different levels of virus resistance were selected for further investigation, namely, monocultures 2090-2 (○) and 2090-BD5 (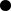) derived from *E. huxleyi* 2090, and monocultures Rec-17 (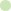), Rec-32 (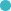), and Rec-53 (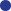) derived from *E. huxleyi* 2090-Rec. (E) Cell abundance of virus-inoculated (solid lines) and non-inoculated (dashed lines) *E. huxleyi* 2090 (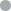) cultures and *E. huxleyi* 2090-derived monocultures. (F) Cell abundance of *E. huxleyi* 2090-Rec (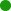) and virus-inoculated (solid lines) and non-inoculated (dashed lines) *E. huxleyi* 2090-Rec-derived monocultures. (G) Virus production in virus-inoculated *E. huxleyi* 2090 and 2090-derived monocultures. (H) Virus production in *E. huxleyi* 2090-Rec and virus-inoculated *E. huxleyi* 2090-Rec-derived monocultures. Values in E–H are presented as mean ± SD (*n* = 3). ^****^P-value <  0.0001 (two-tailed nonparametric Mann–Whitney test). Scheme was created with Biorender.com.

To explore the heterogeneity in virus resistance within the recovered coexisting *E. huxleyi* population compared with the ancestor strain, a single-cell sorting approach was applied. Monoculture collections were generated by sorting single cells originating from either coexisting or susceptible cultures into well plates (Fig. 3A). In total, 74 single cells from *E. huxleyi* 2090-Rec and 123 single cells from the ancestor strain *E. huxleyi* 2090 formed viable growing monocultures. These monoculture collections represent the cell heterogeneity of the population from which they were derived. Therefore, differences between individual monocultures reflect the cell-to-cell phenotypic heterogeneity within the original cell population. To test for the presence of viral particles and thus of possible host–virus coexistence, the abundance of EhV in the medium of all monocultures was measured by qPCR using primers for the EhV *mcp* gene (Fig. S6). Viruses were not detected in any of the monocultures derived from *E. huxleyi* 2090-Rec. Subsequently, all monocultures were phenotyped for virus resistance by monitoring cell abundances (based on chlorophyll fluorescence) and measuring virus production 6 days following the addition of EhV-201 (Fig. 3B and C).

The mean chlorophyll fluorescence of *E. huxleyi* 2090-Rec-derived monocultures was two orders of magnitude higher than that of *E. huxleyi* 2090-derived monocultures (*P*-value <  0.0001, 1.5 × 10^3^ vs. ~10 A.U., Fig. 3B), reflecting their resistance to viral infection. Accordingly, the mean virus production of monocultures derived from *E. huxleyi* 2090 at 6 dpi was ~4-fold higher than that of the *E. huxleyi* 2090-Rec-derived monocultures (*P*-value <  0.0001, 6.5 × 10^8^ vs. 1.5 × 10^8^ viral particles ml^−1^, Fig. 3C). Furthermore, the heterogeneity in virus production was larger in monocultures derived from *E. huxleyi* 2090-Rec compared with monocultures derived from *E. huxleyi* 2090, with a CV of 99.7% vs. 39.1%, respectively. Similarly, *E. huxleyi* 2090-Rec-derived monocultures showed a high diversity in chlorophyll fluorescence, whereas the majority of *E. huxleyi* 2090-derived monocultures were dead, with only two viable monocultures that showed a chlorophyll fluorescence of >10^2^ A.U. These results indicate that *E. huxleyi* 2090-Rec comprises a highly heterogeneous population of cells that vary in their susceptibility, whereas *E. huxleyi* 2090 is comprised of a mostly homogeneous population of susceptible cells. The concomitant occurrence of resistant monocultures having high chlorophyll fluorescence and low viral production, and monocultures with lower virus resistance, having low chlorophyll fluorescence and high viral production, provides evidence for phenotypic heterogeneity (Fig. 3D). The chlorophyll fluorescence and virus production of *E. huxleyi* 2090-Rec monocultures were negatively correlated (*r* = −0.78, *P*-value <  0.0001). These results suggest that the coexisting *E. huxleyi* 2090-Rec population is composed of cells with phenotypes across a continuum of susceptibility rather than following the classical binary definition of either susceptible or resistant. The presence of two monocultures from the susceptible ancestor *E. huxleyi* 2090, which were resistant to viral infection, highlights that viral resistance can also emerge from susceptible populations in the absence of viral pressure. This observation highlights that small seed subpopulations of resistant cells may occur in susceptible populations.

To further characterize the resistant cells that co-occur in the *E. huxleyi* 2090-Rec population, several monocultures with distinct resistance phenotypes were selected. The monocultures Rec-17, Rec-32, and Rec-53 were selected as they span the resistance-susceptibility-continuum observed in the 2090-Rec population, with Rec-17 being the most susceptible and Rec-53 being the most resistant (Fig. 3B–D). In addition, two monocultures derived from the susceptible strain *E. huxleyi* 2090 were selected: 2090-BD5, as an innate resistant monoculture, and 2090-2, as a representative susceptible monoculture (Fig. 3B–D). The selected monocultures were picked from the 96-well plates, grown in larger volumes, and inoculated with EhV-Rec. The infection dynamics were monitored at high temporal resolution by measuring the abundance of cells and virus particles (Fig. 3E–H). Virus-inoculated cultures of monoculture 2090-2 demonstrated growth arrest at 2 dpi and most of the population lysed within 7 dpi, similar to the ancestor *E. huxleyi* 2090 (Fig. 3E). This was coupled with high virus production in both *E. huxleyi* 2090 and 2090-2 starting from 2 dpi (~1 × 10^8^ viral particles ml^−1^, Fig. 3G). In contrast, virus-inoculated cultures of 2090-BD5, Rec-17, Rec-32, Rec-53, and 2090-Rec did not exhibit an intense population decline in parallel to the production of viruses (Fig. 3E–H). Moreover, monocultures 2090-BD5, Rec-53, Rec-32, and Rec-17 displayed growth together with virus production, suggesting the existence of a fraction of susceptible cells within their population. We confirmed their phenotypic heterogeneity through single-cell resistance screening, thus demonstrating the ability of individual cells to generate diverse populations (Fig. S7). Themaximum viral production (MVP, Fig. S8), maximum carrying capacity (CC, Fig. S9), and growth rate (*μ*, Fig. S10) of all cultures were computed to characterize the *E. huxleyi*–EhV infection dynamics and differentiate the levels of virus resistance of these strains. The MVP varied across the virus-containing monocultures: Rec-17 and 2090-2 had a similar MPV to that of the ancestor *E. huxleyi* 2090 (1.6 × 10^8^ viral particles ml^−1^), whereas the other monocultures had lower MPV values compared with *E. huxleyi* 2090. Rec-32 was ~70% lower (0.5 × 10^8^ viral particles ml^−1^), Rec-53 was ~96% lower (7 × 10^6^ viral particles ml^−1^), 2090-BD5 was 99.6% lower (1 × 10^6^ viral particles ml^−1^), and the recovered coexisting *E. huxleyi* 2090-Rec was ~88% lower than *E. huxleyi* 2090 (2 × 10^7^ viral particles ml^−1^, Fig. S8). In the presence of viruses, the monocultures Rec-53, 2090-BD5, and 2090-Rec demonstrated high CCs (about 3 × 10^6^ cells ml^−1^), Rec-17 and Rec-32 displayed lower CCs (2 × 10^6^ cells ml^−1^), whereas the susceptible *E. huxleyi* 2090 and 2090-2 showed a low CC (<1 × 10^6^ cells ml^−1^), suggesting that a high CC in the presence of viruses illustrates a high resistance level (Fig. S9A). Furthermore, the non-inoculated monocultures Rec-17, Rec-32, Rec-53, and 2090-BD5 displayed a lower CC and *μ* than non-inoculated *E. huxleyi* 2090 and 2090-2, highlighting low CC and growth rate as a cost of resistance (Figs 3E and F and S9B and S10A). Taken together, by assessing diverse phenotypes originating from the same isogenic population, we propose that *E. huxleyi* can generate cells with different resistance levels. Characterizing virus resistance at single-cell resolution unveiled substantial cell-to-cell heterogeneity within the *E. huxleyi* 2090-Rec population. Cells that share the same genetic background can have different phenotypes leading to complex population dynamics. While less resistant cells may be infected and even lysed by the virus, more resistant cells will continue to multiply. Thereby a new generation of a heterogeneous population is generated with individual cells that vary in their levels of resistance.

### Benefits of host–virus coexistence during intraspecies competition

**Figure 4:**
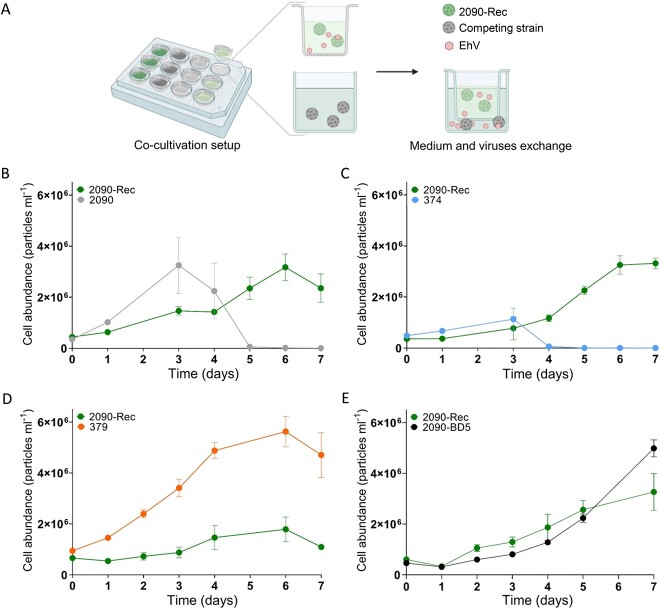
Viral production in the coexisting *E. huxleyi* 2090-Rec population is advantageous during competition with other, susceptible host strains. (A) Schematic representation of the cocultivation setups. Couples of *E. huxleyi* strains were cocultured in 24-well plates separated by a 1-μm membrane, allowing the exchange of nutrients, metabolites, and viruses, but preventing the mixing of algal cells. Cell abundances of the coexisting *E. huxleyi* 2090-Rec during cocultivation with *E. huxleyi* 2090 (B), 374 (C), 2090-BD5 (D), and 379 (E). Strain 2090-Rec was grown in the insert and the competing strains within the surrounding wells. Values in B–E are presented as mean ± SD (*n* = 3). Scheme was created with Biorender.com.

To assess possible ecological consequences of *E. huxleyi*–EhV coexistence in mixed natural bloom populations, we conducted a series of cocultivation assays simulating intraspecies competition between *E. huxleyi* strains that differ in their virus resistance. The coexisting *E. huxleyi* 2090-Rec was cocultured with either susceptible strains (*E. huxleyi* 2090 or 374), a fully resistant strain (*E. huxleyi* 379), or *E. huxleyi* 2090-BD5 that is overall resistant while showing minor viral production after EhV inoculation. The cultures were separated by a 1-μm pore size membrane, allowing the exchange of viruses, nutrients, and metabolites, but not of algal cells (Fig. 4A). During cocultivation of *E. huxleyi* 2090-Rec with its susceptible ancestor *E. huxleyi* 2090, *E. huxleyi* 2090 outgrew the coexisting population in the first three days, reflecting a higher growth rate and a possible trade-off for resistance. However, in the subsequent days, the *E. huxleyi* 2090 population rapidly declined because of infection by the viruses (EhV-Rec) released from *E. huxleyi* 2090-Rec (Fig. 4B). These results emphasize how the slow-growing *E. huxleyi* 2090-Rec outcompetes the fast-growing susceptible *E. huxleyi* 2090 by continuous release of infectious virions. EhV produced by the coexisting population serves as a weapon against the susceptible strain leading to its decline and more viral progeny (Fig. S11A). Similar results were observed by coculturing *E. huxleyi* 2090-Rec with the susceptible strain *E. huxleyi* 374 (Figs 4C and S11B). On the other hand, when cocultured with the resistant strain *E. huxleyi* 379, *E. huxleyi* 2090-Rec was outgrown (Fig. 4D). *Emiliania huxleyi* 379 reached a substantially higher cell density compared with *E. huxleyi* 2090-Rec (5.6 × 10^6^ and 1.8 × 10^6^ cells ml^−1^, respectively), suggesting that heterogeneity and host–virus coexistence is not a favorable competition strategy against a homogenous resistant algal population. Nevertheless, the abundance of resistant strains is typically extremely low in *E. huxleyi* blooms [35].

When *E. huxleyi* 2090-Rec was cocultured with *E. huxleyi* 2090-BD5, the coexisting *E. huxleyi* 2090-Rec grew to similar cell densities as *E. huxleyi* 2090-BD5 (Fig. 4E). This result indicates that there is no fitness advantage for either strain when co-occurring in the same population and that EhV-Rec produced by *E. huxleyi* 2090-Rec (Fig. S11C) had no significant effect on the growth of *E. huxleyi* 2090-BD5 (*P*-value =  0.6986). Furthermore, in the absence of viruses, the susceptible strain *E. huxleyi* 2090 outcompeted the resistant *E. huxleyi* 2090-BD5 (Fig. S12F), highlighting the cost of resistance in a virus-free environment. This cost of resistance may explain why so few resistant cells were isolated from *E. huxleyi* 2090.

Taken together, the heterogeneous *E. huxleyi* 2090-Rec population represents a mutualistic host–virus interaction. The coexistence with a lytic virus is advantageous for *E. huxleyi* during intraspecies competition via the proliferation of viruses by a minor subpopulation of susceptible cells, whereas the overall resistant cell population continues to grow. Moreover, host–virus coexistence emerged in several other *E. huxleyi*–EhV interactions (Fig. S13). This includes well-studied strains and several strains that we recently isolated from an *E. huxleyi* bloom in Norway. These results suggest host–virus coexistence can be a common strategy during *E. huxleyi* blooms.

## Discussion

Host extinction is an undesirable outcome for viruses because of their dependency on the host’s cellular machinery for replication. Previous studies have showed that viruses with lysogenic or chronic lifestyles can efficiently coexist with their host [42]. Some bacterial and archaeal lysogenic phages have extended the coexistence with their host by enhancing host fitness through integrating into the genome and introducing beneficial genes to the host [43-45].

In contrast, observations of coexistence between lytic viruses and eukaryotic hosts are limited and their underlying mechanisms underexplored. The dilemma of how a host and a lytic virus coexist becomes even more complex when the virus infects a bloom-forming alga, whose availability at high densities is limited to ephemeral bloom events [46, 47].

### Heterogeneity within the host population is a driving force for host–virus coexistence

When the entire *E. huxleyi* population shares uniform susceptibility to the virus, an encounter with EhV should lead to the lysis of all cells and host extinction. Conversely, if all cells are uniformly resistant, there will be no virus progeny leading to virion decay and virus extinction. Host populations exhibiting these binary characteristics will not be able to maintain coexistence with a lytic virus. The observed phenotypic heterogeneity in *E. huxleyi* led us to hypothesize that host–virus coexistence is driven by the continuous formation of heterogeneous cells characterized by different resistance levels. The emergence of a more resistant cell (i.e. *E. huxleyi* 2090-BD5) within a susceptible population (*E. huxleyi* 2090) in the absence of viral pressure (Fig. 3D) implies that heterogeneity within the susceptible population can prevent its extinction by EhV infection. These resistant cells can survive viral infection to form a new population (Fig. 1B). We suggest that the continuous formation of heterogeneous cells with different resistance levels also caused stable *E. huxleyi*–EhV coexistence in the recovered population (2090-Rec). Because of the continuous generation of a minor subpopulation of susceptible cells (Fig. 2B), the virus can propagate and lyse its host, whereas most resistant cells continue to multiply. Every division generates new heterogeneous cells that include a new fraction of susceptible cells. In this context, no susceptible cells (susceptible as the majority of 2090 cells) were isolated from the coexisting 2090-Rec population (Fig. 3D), suggesting that susceptible cells are lysed before or after sorting thus preventing the formation of a monoculture. Furthermore, none of the monocultures produced viruses, indicating that all the 2090-Rec derived single cells that yielded monocultures upon sorting were resistant to the virus. These results highlight the necessity of an assortment of cells with susceptible and resistant phenotypes for a lytic virus to coexist with its host. Furthermore, the screening for resistance of a population derived from a single cell (Fig. S7) implies that individual cells can develop phenotypic heterogeneity within only a few generations. All cells within a monoculture share the same genetic background, pointing out that virus resistance may be a trait with high plasticity.

In other microbial systems, host–virus coexistence was shown to be based on the limited infection of one of two host subpopulations. An early observation from 1945 [48] pointed out that “reverse mutations” can cause a small number of resistant bacterial cells to become susceptible to lytic phage infection. Similarly, studies of the host–virus coexistence in *Escherichia coli* [9, 49] and the marine microalga *Ostreococcus mediterraneus* [6] demonstrated that this interaction is based on two subpopulations with contrasting phenotypes with the underlying mechanism being attributed to gene mutations and genome rearrangements. The source of the observed cell-to-cell heterogeneity in the coexisting *E. huxleyi* population remains elusive. Possible explanations for the emerged resistant phenotypes may involve (i) genetic modifications, including random mutations [49], chromosome rearrangements [50], mobile genetic elements such as transposons [51, 52], and horizontal gene transfer [53], as well as (ii) nongenetic modifications, such as methylations that impact epigenetics [54, 55] or programmed transcriptional responses [28, 56]. The isolation of monoculture 2090-BD5 from an uninfected *E. huxleyi* 2090 culture indicates that such molecular changes can also occur spontaneously. Other processes may mediate host–virus coexistence on the population level. These processes may work in parallel to the formation of cell-to-cell heterogeneity. This includes the release of molecules during cell lysis that prevent virus attachment [21], and the presence of a “numerical refuge” by which low densities of susceptible cells avoid extinction through a low probability of virus encounter [57].

### Continuum of virus resistant phenotypes as a bet-hedging strategy

**Figure 5:**
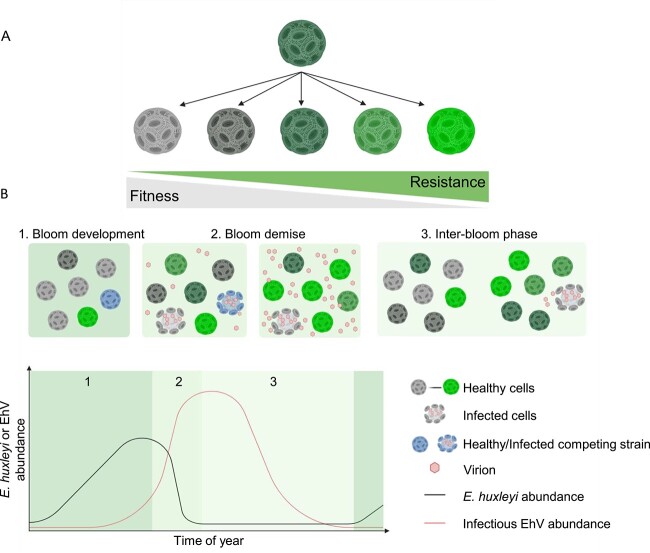
Conceptual model describing how cell-to-cell phenotypic heterogeneity in *E. huxleyi* response to viral infection can drive bloom succession. (A) An individual *E. huxleyi* cell can generate cells with diverse resistant levels stretching along a continuum of resistance to susceptibility. Each phenotype possesses a trade-off between fitness and virus resistance resulting in a heterogeneous population. (B) *E. huxleyi* phenotypic heterogeneity in response to viral infection over time. Environmental stresses and virus abundance act as a selection force for the best-fitted *E. huxleyi* phenotypes. 1. During bloom development, susceptible fast-growing competitive cells compose the majority of the *E. huxleyi* bloom because of higher fitness and low virus abundance. 2. As a dominant mortality agent for bloom demise, EhV lyses susceptible cells, thereby altering the ratio between different resistant phenotypes. The cells that survived viral infection are composed of the preexisting resistant seed population. These cells can multiply under high virus load and can give rise to a new generation of heterogeneous population. 3. During interannual bloom phase, *E. huxleyi* may maintain coexistence with EhV at the population level by generating a new fraction of susceptible cells that will be infected by EhV. In the case of ineffective contact rate between susceptible cells and EhV, susceptible cells with neglectable cost of resistance may out-grow resistant cells. The latter will remain as a small seed population until the next bloom demise. Continuous production of susceptible cells by resistant cells in heterogeneous populations may serve as a weapon by the proliferation and released of EhV that can lyse co-occurring competing *E. huxleyi* strains. Model was created with Biorender.com.

Mapping virus resistance at a single-cell resolution revealed a spectrum of resistance levels within the *E. huxleyi* population (Fig. 5A). As described by the “Kill the Winner” hypothesis, virus resistance comes with a cost [58], such as lower nutrient assimilation [9, 49], higher susceptibility to viral infection by other viruses [53, 59], or reduced growth rate [8, 26, 60], as was observed also in this study (Fig. S10). Thus, the high cell-to-cell heterogeneity may provide a bet-hedging strategy to cope with changing environments [61]. A reservoir of diverse cell types with different degrees of susceptibility will allow the best-fitted phenotype to be selected under a given environment. The many levels of resistance may serve as an adaptive strategy to optimize the tradeoff between virus resistance and acclimation to varying levels of environmental stresses. We hypothesize that the presence or absence of viruses shapes the population composition by promoting either resistant, less competitive (lower *μ*), or susceptible, highly competitive cells, respectively. We suggest that the selection pressure of the environment is the guiding principle of the phenotypic variability in population composition of the naïve *E. huxleyi* 2090 population compared with the coexisting *E. huxleyi* 2090-Rec population. For example, the naïve *E. huxleyi* 2090 population consists of a majority of susceptible cells (Fig. 3D), whereas the *E. huxleyi* 2090-Rec population consists of a majority of resistant cells (Figs 2C and 3D). The *E. huxleyi* 2090-BD5 strain is a rare resistant strain isolated from an uninfected *E. huxleyi* 2090 population ('innate resistance'), grows slower than *E. huxleyi* 2090 when cocultured in the absence of viruses (Figs S10 and S12F), illustrating how susceptible cells can take over the population without viral pressure. Accordingly, in the absence of viruses, some subcultures of the resistant 2090-BD5 monoculture displayed loss of the resistant phenotype over several months (data not shown). Similarly, loss of resistance was observed occasionally for subculture of other resistant monocultures, namely, Rec-53, Rec-17, and Rec-32, but not 2090-Rec, which maintains a constant selection for resistant cells by producing viruses. Our results suggest that susceptible cells have an advantage in virus-free environments (Fig. S12F), whereas cells with a high resistance level have an advantage in an environment with a high viral load. Nonetheless, producing a heterogeneous population with a fraction of susceptible cells possesses an additional advantage. The production of susceptible cells supports EhV proliferation, providing an effective weapon of infectious virions that may lyse co-occurring strains evolving during bloom succession (Fig. 4B and C). Our results thereby show a new interaction between algae and their lytic viruses. The cost for *E. huxleyi* coexistence with EhV is the death of the susceptible fraction of the population and proliferation of only the resistant cells, and the risk of competing against a strain with a higher resistance level (2090-Rec vs. 2090-BD5 and 2090-Rec vs*.* 379). However, resistant cells are rare during bloom events [35]. The *E. huxleyi*–EhV coexistence simultaneously demonstrates the lysis of some cells with beneficial consequences for the entire population.

The finding of multiple resistance phenotypes stretching on a continuum [62, 63] offers a novel perspective on the binary classification of susceptibility or resistance. This continuum adds complexity to the classical view of species and strain-specific networks of host–virus interactions. We introduce a higher resolution of host specificity implying that the infection outcome may vary depending on the individual cell. The cellular mechanisms that can account for different levels of resistance against viral infection between individual *E. huxleyi* cells are currently unknown and may comprise various strategies. Previous studies indicated that virus resistance can be associated with morphological changes in the formation of organic scales on the *E. huxleyi* cell surface [26] and unique lipid composition [35]. The recently identified lipid biomarkers for resistance were not identified in *E. huxleyi* 2090-BD5 and 2090-Rec (data not shown), suggesting that their resistance did not arise from membrane alterations. Gaining various levels of virus resistance may also be tightly linked to cell metabolism. Viral infection is highly affected by the cellular physiological state because of the dependency on cellular metabolic pathways and resources for viral progeny [42]. As a giant virus with a large burst size [64-66], EhV has a high metabolic demand that must be met by the host cell. This offers numerous possibilities by which remodeling of host metabolism can lead to varying levels of cell resistance with metabolic costs. In addition, differential expression of metabolic genes was shown to be associated with resistance or susceptibility in various *E. huxleyi* strains [28, 35, 67]. Lastly, various defense mechanisms may lead to resistance, as was found in prokaryote genomes that carry a wide arsenal of antiviral systems [68]. Utilization of more than one defense system often has a complementary, overlapping, or cumulative effect against different viral species [68]. Taken together, further investigation is needed to identify antiviral defense mechanisms in *E. huxleyi* and their distribution among cells in the population to explain the continuum of resistance levels.

### The ecological significance of phenotypic heterogeneity in response to viral infection

*Emiliania huxleyi*–EhV interactions in the ocean are driven by genetic and phenotypic diversity. While the genetic diversity of *E. huxleyi* and EhV strains in natural blooms has been already reported [14, 69, 70], it remains challenging to account for phenotypic diversity in the natural environment. We established an experimental setup to ask fundamental ecological questions regarding host–virus coexistence, using as a model system an *E. huxleyi* culture and its derived monocultures with diverse phenotypes. It is likely that bloom termination by EhV induces a strong selection pressure for the survival of rare preexisting resistant *E. huxleyi* subpopulations similar to the resistant strain 2090-BD5 (Fig. 3D), which can act as seed populations for subsequent blooms (Fig. 5). Although the tools to quantify cells of various resistant phenotypes during and after bloom events are still required, our results suggest that following virus-induced bloom demise, surviving resistant cells may form coexistence with EhV by generating a new susceptible daughter population similarly to the laboratory cultures (Figs 1B and S13). Bottom-up conditions, such as nutrient limitation, or top-down control by other mortality agents of *E. huxleyi* probably limit the growth of a new population. However, under conditions of effective host–virus contact rates, a coexistence of *E. huxleyi* and EhV could occur, which benefits both the alga and the virus by increasing algal competitiveness and allowing prolonged association of EhV with its host, particularly during the long periods of interannual blooms. If the probability to encounter a virus is extremely low, our findings indicate that slow-growing resistant cells will be outcompeted by cells that are typically bloom-forming, i.e. opportunistic, competitive, and virus-susceptible cells [27, 71] (Fig. S12F). This illustrates that a population of resistant cells that survived a virus attack could be outgrown by susceptible cells setting the stage for the next bloom event (Fig. 5B). This observation may contribute to previous reports showing high seasonal stabilities of marine microbial and viral communities [72, 73], and the reoccurrence of the same *E. huxleyi* and EhV genotypes in a multi-annual manner [74].

The use of single-cell analysis to characterize virus resistance at single-cell resolution revealed a substantial cell-to-cell heterogeneity. The comparison of *E. huxleyi* cells with identical genetic backgrounds and distinct resistant phenotypes provides a promising approach for future discoveries of ecologically relevant antiviral strategies. We propose that the generation of phenotypic heterogeneity is pivotal for the *E. huxleyi*–EhV arms race in the natural environment and allows the emergence of better adapted cells to survive. Therefore, host heterogeneity is a driving force to enable the coexistence of *E. huxleyi* and EhV in the ocean.

## Supplementary Material

Joffe_et_al_ISME_050224_SI_wrae038

## Data Availability

Data supporting the findings of this study are available in the paper and its Supplementary Information. Mass spectral raw data (MS^E^) was deposited in the EMBL­EBI MetaboLights repository with the identifier MTBLS1956 (www.ebi.ac.uk/metabolights/MTBLS1956).

## Preprint

This manuscript was published as a preprint on bioRxiv (https://doi.org/10.1101/2023.10.03.560477). The copyrights are held by the authors only, granting bioRxiv only a license to display the preprint in perpetuity. No reuse is allowed without permission.
